# Predicting lymphovascular space invasion in early-stage cervical squamous cell carcinoma using heart rate variability

**DOI:** 10.3389/fonc.2025.1562347

**Published:** 2025-07-21

**Authors:** Junlong Fang, Ming Liu, Zhijing Song, Yifang Zhang, Bo Shi, Jian Liu, Sai Zhang

**Affiliations:** ^1^ School of Clinical Medicine, Bengbu Medical University, Bengbu, Anhui, China; ^2^ Department of Gynecologic Oncology, First Affiliated Hospital, Bengbu Medical University, Bengbu, Anhui, China; ^3^ School of Medical Imaging, Bengbu Medical University, Bengbu, Anhui, China

**Keywords:** cervical cancer, cervical squamous cell carcinoma, heart rate variability, lymphovascular space invasion, autonomic nervous system

## Abstract

**Background:**

Accurate preoperative assessment of lymphovascular space invasion (LVSI) in patients with early-stage cervical squamous cell carcinoma (ECSCC) is clinically significant for guiding treatment decisions and predicting prognosis. However, current LVSI assessment of ECSCC mainly relies on the invasive method of pathological biopsy, which needs to be further improved in terms of convenience. The main objective of this study is to verify the value of preoperative heart rate variability (HRV) parameters in predicting ECSCC LVSI.

**Methods:**

A total of 79 patients with ECSCC confirmed by postoperative pathology were enrolled in this study at the Department of Gynecologic Oncology of the First Affiliated Hospital of Bengbu Medical University. Patients were classified as LVSI-positive (LVSI+) or LVSI-negative (LVSI-) based on pathological examination. Preoperative 5-minute electrocardiogram (ECG) data were collected from all patients, and their HRV parameters were analysed, including 7 time-domain parameters, 5 frequency-domain parameters, and 2 nonlinear parameters. Ten HRV features were selected through univariate analysis, and a logistic model was constructed using age, body mass index, menopausal status, and mean heart rate to predict LVSI status. The model performance was evaluated by the area under the receiver operating characteristic curve (AUC), accuracy, precision, sensitivity, and specificity.

**Results:**

The constructed model showed good predictive performance, with an AUC of 0.845 (95% CI: 0.761 - 0.930), sensitivity of 0.871, specificity of 0.750, precision of 0.690, and accuracy of 0.747.

**Conclusions:**

The Logistic model constructed based on HRV features has a relatively good diagnostic performance in predicting the LVSI status of ECSCC, but further research is still needed through larger datasets, more features, and the combination of machine learning models.

## Introduction

Cervical cancer (CC) is the fourth most common cancer among women globally, with 661,000 new cases and 348,000 deaths worldwide in 2022 (1). Widespread adoption of mass screening and preventive HPV vaccines has led to earlier diagnoses of CC in female patients (2). The 5-year overall survival rate for each stage of CC is 67%, with 91% for early stage, 60% for locally advanced stage, and 19% if accompanied by distant metastasis (3). Lymphovascular space invasion (LVSI) refers to the presence of tumor cells in the lymphatic vessels and/or small capillaries outside the primary tumor, in the lymphatic and/or vascular spaces (4). Previous studies have shown that LVSI is a high-risk factor for regional lymph node metastasis in early-stage CC patients and is closely related to local recurrence and distant metastasis after surgical treatment (5–9). The Gynecologic Oncology Group (GOG) classifies LVSI, stromal invasion, and tumor size as moderate-risk factors for prognosis and adjuvant therapy decisions, which called “Sedlis criteria” (10). The 2024 National Comprehensive Cancer Network (NCCN) CC Clinical Practice Guidelines for the United States also emphasize (11), LVSI plays a crucial role in selecting the treatment plan for early cervical cancer, directly affecting the prognosis of cervical cancer patients. Under the condition of meeting the “Sedlis criteria”, patients should undergo pelvic adjuvant radiotherapy ± chemotherapy (12).

Pathological examination is the gold standard for diagnosing LVSI, and immunohistochemistry can help improve the accuracy of the diagnosis (13). Specimens can be obtained from cervical biopsies, loop electrosurgical excision procedures (LEEP), cold knife conizations (CKC), and hysterectomies. Hysterectomy specimens yield diagnostic information only intraoperatively or postoperatively, creating a time lag that precludes their use in preoperative therapeutic planning. Specimens obtained from cervical biopsy, LEEP, and CKC have lower sensitivity for detecting LVSI (14). In order to find more effective preoperative LVSI prediction methods, an increasing number of scholars are turning their attention to imaging genomics. A recent meta-analysis indicates that imaging genomics based on magnetic resonance imaging (MRI) has good predictive performance for LVSI before CC surgery, with an overall sensitivity of 0.83 and a specificity of 0.74 (15). However, there is a lack of uniform standards for acquiring and processing imaging data, which affects the consistency and comparability of feature extraction, which is the core of imaging genomics (16). Therefore, the use of imaging genomics for predicting CC LVSI in clinical practice lacks stability.

Autonomic nervous system (ANS) plays a crucial role in the regulation of the tumor microenvironment. It can directly control the tumor microenvironment through neurotransmitters such as norepinephrine and acetylcholine. For instance, by suppressing the immune function of natural killer cells, promoting angiogenesis and enhancing the permeability of the endothelial barrier, thereby increasing the invasive and metastatic potential of tumors (17, 18). Heart rate variability (HRV), as a non-invasive and quantitative tool for evaluating the function of ANS (19), has been confirmed to be closely related to the disease progression of various malignant tumors (20), including CC (21), breast cancer (22), colorectal cancer (23), gastric cancer (24) and small cell lung cancer (25). As studies in prostate cancer and gastric cancer have shown, a reduction in HRV is significantly correlated with high expression of vascular endothelial growth factor (VEGF) and an increased risk of distant metastasis, suggesting that autonomic nerve dysfunction may accelerate tumor dissemination through the angiogenesis pathway (26). Our previous research found that HRV is an independent predictor of CC lymph node metastasis (21). According to the theory of metastatic cascade, LVSI is a key pathological process for tumor cells to achieve metastasis (27). Its essence is the process by which tumor cells penetrate the vascular endothelial barrier and enter the circulatory system (4). However, no study has yet explored the relationship between HRV and LVSI in CC. The main purpose of this article is to verify the value of HRV parameters in preoperative prediction of LVSI in early-stage cervical squamous cell carcinoma (ECSCC).

## Methods

### Subjects

In November 2020 to September 2023, 159 patients with CC confirmed by pathological examination were included in this study at the Department of Gynecologic Oncology of the First Affiliated Hospital of Bengbu Medical University. Inclusion criteria: (1) Primary patients who have not received surgical treatment, radiotherapy or neoadjuvant chemotherapy.; (2) All patients included in the groups were confirmed to have cervical squamous cell carcinoma after pathological histological examination; (3) FIGO 2018 staging of IIA1 or lower (the FIGO staging system assessment was completed based on the postoperative pathological examination results). Exclusion criteria: (1) Incomplete clinical and/or data (n=71); (2) ECG quality signal is poor (n=2); (3) Ectopic heartbeats >5% (n=6); (4) Lymph node metastasis has already occurred (n=1). This study was approved by the Ethics Committee of the First Affiliated Hospital of Bengbu Medical University (registration number: 2023-14) and was conducted in accordance with all relevant regulations and guidelines. All participants were informed of the detailed purpose, process, risks, and adverse effects of the experiment before data collection and signed informed consent forms.

### Measurement outcome

**Figure 1:**
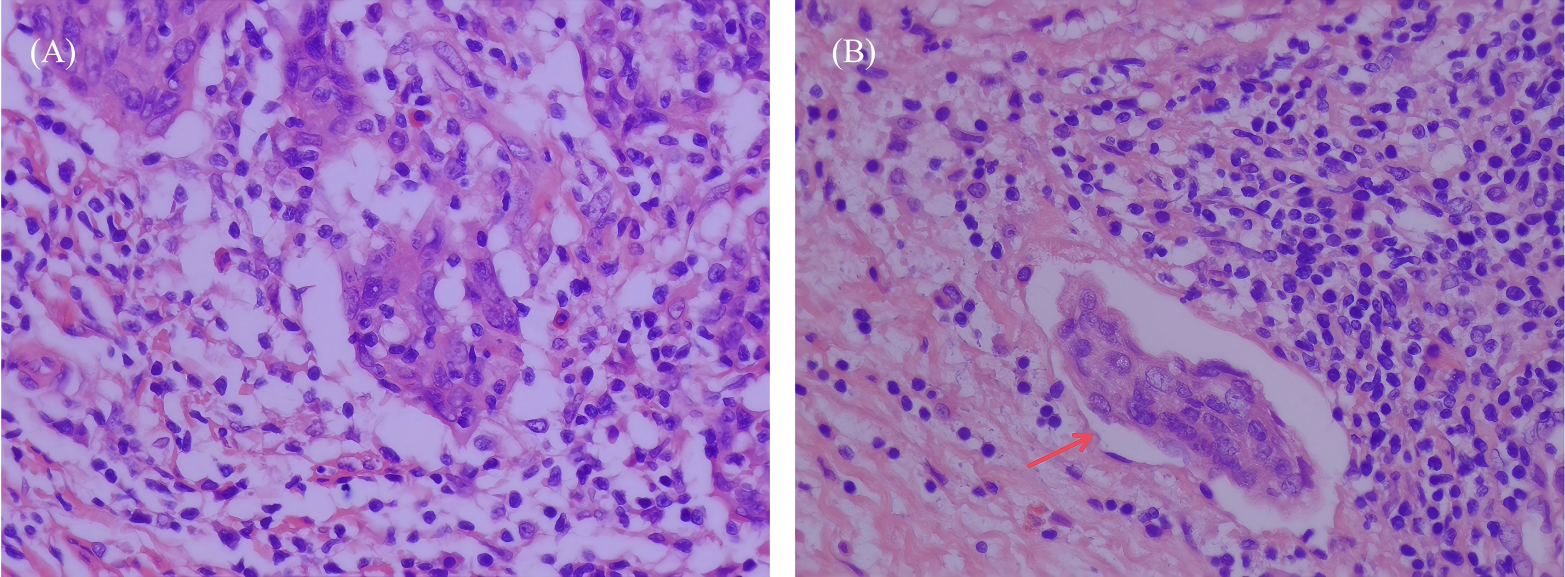
H&E stained images of LVSI negative and positive sections in early cervical squamous cell carcinoma tissues (×400 magnification, representative areas selected from postoperative pathological specimens). **(A)** LVSI-negative section, no tumor cell infiltration of lymphovascular spaces was observed. **(B)** LVSI-positive section, arrows indicate tumor cell infiltration of LVSI, presenting as tumor cell clusters located within the lumen of vascular endothelium.

This study prepared specimens of hysterectomy and prepared sections for examination. Using hematoxylin and eosin (H&E) staining, LVSI-positive was defined as at least one cluster of tumor cells was clearly visible in the normal lymphatic or vascular spaces around the tumor using an optical microscope,otherwise LVSI-negative (13) (**Figure 1**). For complex cases, immunohistochemistry (IHC) was used, which utilised specific markers to distinguish true LVSI from artifacts. To enhance the recognition rate of LVSI, two senior pathologists independently observed the slides without knowing the patient’s clinical information and jointly evaluated the results. If the results were inconsistent, we would resolve the dispute through discussion or by introducing the opinion of a third senior pathologist.

### ECG collection and heart rate variability analysis

Use a single-lead miniature electrocardiogram (ECG) recorder (Healink-R211B, HeaLink Ltd., Bengbu, China) to record the patient’s ECG in the supine position 5 minutes before surgery. The ECG sampling rate is 400 Hz, the bandwidth is 0.6–40 Hz, and the lead configuration is V6 lead. During the test, it is required to have a quiet environment, and the patient is instructed to remain quiet and breathe evenly during the ECG measurement. The ECG signals are processed using the Pan-Tompkins algorithm to extract the R-R intervals (RRI) time series (28). An adaptive threshold algorithm is used to automatically correct for false positives in the RRI caused by the extraction algorithm, interference, ectopic beats, and arrhythmias (29). Performing HRV analysis on the RRI time series, the time domain parameters include the mean heart rate (Mean HR), the standard deviation of all normal-to-normal intervals (SDNN), the root mean square of successive interval differences (RMSSD), triangular interpolation of normal-to-normal intervals (TINN), RR interval triangular index (RRTI), deceleration capacity (DC), and acceleration capacity (AC); the frequency domain indicators include total power (0-0.4 Hz, TP), very low frequency (0-0.04 Hz, VLF), low frequency (0.04-0.15 Hz, LF), high frequency (0.15-0.4 Hz, HF), and ratio between LF and HF (LF/HF). Nonlinear parameters include approximate entropy (ApEn) and sample entropy (SampEn). Before performing a frequency domain analysis, the RRI is first uniformly resampled (using a cubic spline interpolation method with a sampling rate of 4 Hz). The spectral values are estimated using the Fast Fourier Transform (FFT), and then the Welch periodogram method (window width of 150 seconds, 50% overlapping windows) is used to estimate the power spectral density of the RRI time series. The calculation parameters for entropy are set as follows: delay delay=1, embedding dimension m=2, tolerance r=0.2*SDNN. The above parameters were analyzed using Kubios HRV software (Scientific 4.1.0, https://www.kubios.com, Kubios Oy, Kuopio, Finland).

### Statistical analysis

We used the Shapiro-Wilk test to test the normality of the data. If the data met the normal distribution, the quantitative data were expressed as the mean ± standard deviation. If the data did not meet the normal distribution, the quantitative data were expressed as [M50(P25, P75)]. The comparison between two groups was conducted using the independent sample t-test for normal data and the Mann-Whitney U test for non-normal data; count data was represented by [n(%)] and the comparison between the two groups was conducted using the chi-square test. *P*<0.05 indicates a statistically significant difference. Univariate logistic regression was used to screen HRV parameters with statistical differences to establish a model, and a combined model was constructed by integrating clinical parameters. Draw a receiver operating characteristic (ROC) curve for the subjects and calculate the area under the receiver operating characteristic curve (AUC), accuracy, sensitivity, specificity, and precision to evaluate the predictive performance of the model. The statistical analyses were conducted using SPSS Statistics 27.0 and GraphPad Prism 9.5.1.

## Results

### Baseline characteristics

According to the inclusion and exclusion criteria, a total of 79 CC patients were included in this study. Among them, 31 patients exhibited LVSI-positive status, while 48 patients were LVSI-negative. The comparison of the clinical characteristics and the HRV parameters between the LVSI-positive and the LVSI-negative subjects is shown in **Table 1**. The median age of patients in both groups was 55 years, and there was no statistical difference in body mass index (BMI) and menopausal status. Compared with LVSI-negative patients, LVSI-positive patients had significantly higher HRV parameters such as SDNN, RMSSD, RRTI, TINN, DC, TP, VLF, LF, and HF, while AC was significantly lower, all with statistical significance (*P* < 0.05)(**Table 1**).

**Table 1:** Clinical characteristics and HRV parameters of patients.

Characteristics	LVSI-positive (*N* = 31)	LVSI-negative (*N* = 48)	*P*-value
Age (years)	55.0 (44.0, 60.0)	55.0 (50.3, 60.8)	0.475
BMI (kg/m^2^)	24.8 ± 3.1	24.6 ± 3.4	0.762
Menopausal status			0.773
No	10 (32.3%)	17 (35.4%)	
Yes	21 (67.7%)	31 (64.6%)	
FIGO stage (N, %)			0.587
IA1	0 (0.0%)	1 (2.1%)	
IB1	3 (9.7%)	4 (8.3%)	
IB2	11 (35.5%)	19 (39.6%)	
IB3	9 (29.0%)	18 (37.5%)	
IIA1	8 (25.8%)	6 (12.5%)	
Mean HR (bpm)	68 (64, 79)	69 (66, 74)	0.695
SDNN (ms)	31.2 ± 10.8	24.7 ± 9.4	**0.006**
RMSSD (ms)	19.3 (15.9, 34.0)	14.1 (10.5, 18.9)	**0.001**
RRTI	8.2 ± 2.6	6.8 ± 2.2	**0.008**
TINN (ms)	148.4 ± 49.5	118.9 ± 44.1	**0.007**
DC (ms)	22.0 (17.9, 35.2)	15.3 (11.7, 20.6)	**<0.001**
AC (ms)	-23.6 (-39.2, -18.7)	-16.4 (-22.8, -11.7)	**0.002**
TP (ms^2^)	728 (530, 1429)	422 (219, 806)	**0.001**
VLF (ms^2^)	407 (209, 759)	273 (114, 427)	**0.013**
LF (ms^2^)	120 (80, 257)	75 (49, 121)	**0.001**
HF (ms^2^)	153 (84, 340)	67 (35, 110)	**0.002**
LF/HF	0.92 (0.55, 1.74)	0.97 (0.56, 2.01)	0.779
ApEn	1.15 (1.07, 1.20)	1.14 (1.08, 1.18)	0.707
SampEn	1.59 (1.49, 1.66)	1.55 (1.38, 1.67)	0.325

LVSI, Lymphovascular Space Invasion.

*P-*values with statistical significance are in bold font (*P*<0.05).

### Associations between HRV parameters and LVSI

**Figure 2:**
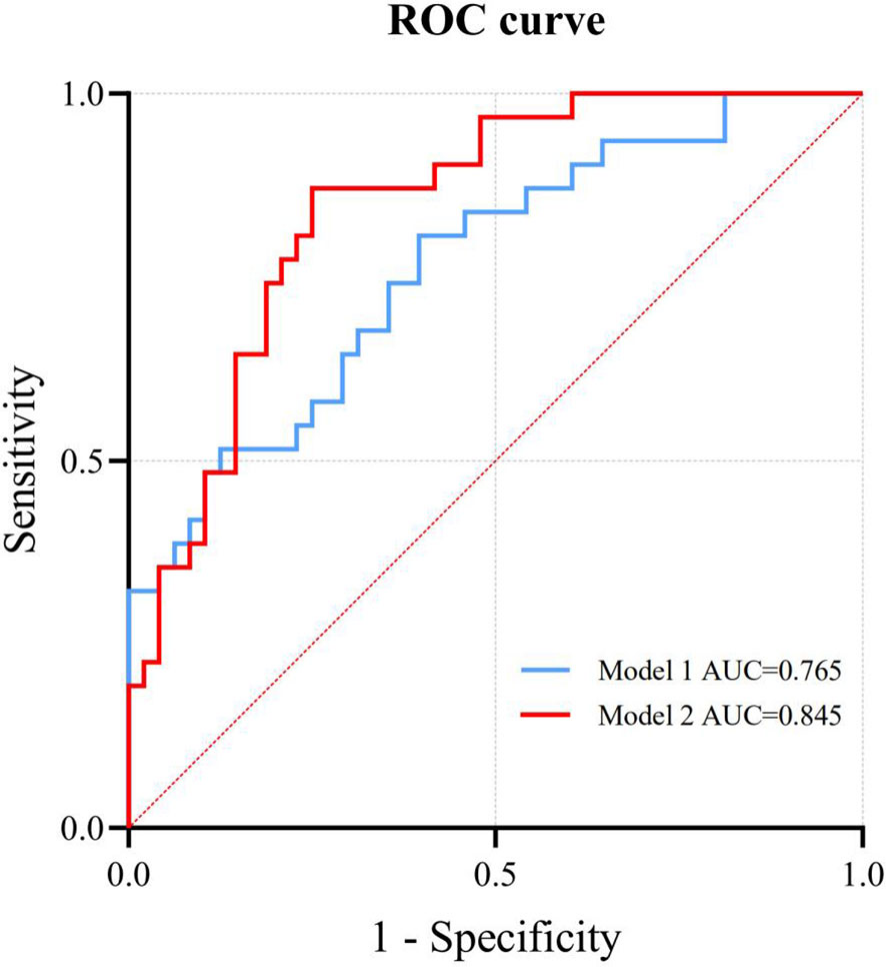
ROC curves for Model 1 and Model 2.

Binary logistic regression analysis shows that SDNN, RMSSD, RRTI, TINN, DC, AC, TP, VLF, LF, and HF are still significantly related to LVSI (*P* < 0.05) (**Table 2**). Among them, SDNN, RMSSD, RRTI, TINN, DC, TP, VLF, LF, and HF are positively correlated with LVSI, while AC is negatively correlated with LVSI. We constructed Model 1 by integrating HRV parameters with statistically significant differences and then combined it with clinical characteristics, including age, BMI, menopausal status, and mean HR, to create Model 2. The relationship between HRV parameters and LVSI was evaluated using ROC curves, with AUC values of 0.765 for Model 1 and 0.845 for Model 2 (**Figure 2**). **Table 3** provides detailed information on the performance of HRV feature parameters, Model 1, and Model 2 in predicting LVSI, including AUC, sensitivity, specificity, precision, and accuracy. Model 1 and Model 2 have better AUC than the single-variable model. For Model 2, the sensitivity was 0.871, the specificity was 0.750, the precision was 0.690, the accuracy was 0.747, and the overall predictive performance was the best.

**Table 2:** Binary logistic regression analysis of the HRV parameters for predicting LVSI.

HRV parameters	OR (95% CI)	*P-*value
SDNN	1.950 (1.170 - 3.250)	**0.010**
RMSSD	1.759 (1.067 - 2.899)	**0.027**
RRTI	1.900 (1.147 - 3.147)	**0.013**
TINN	1.931 (1.163 - 3.208)	**0.011**
DC	1.769 (1.072 - 2.919)	**0.026**
AC	0.551 (0.338 - 0.898)	**0.017**
TP	2.317(1.218 - 4.406)	**0.010**
VLF	1.912 (1.043 - 3.502)	**0.036**
LF	2.610 (1.206 - 5.648)	**0.015**
HF	1.744 (1.018 - 2.988)	**0.043**

OR, Odds Ratio; CI, Confidence Interval.

HRV parameters in the table are normalized by Z-scores.

*P-*values with statistical significance are in bold font (*P*<0.05).

**Table 3:** Risk factors and combined models for predicting LVSI.

Models	AUC (95% CI)	Sensitivity	Specificity	Precision	Accuracy
SDNN	0.677 (0.559 - 0.796)	0.903	0.417	0.500	0.608
RMSSD	0.718 (0.601 - 0.836)	0.806	0.667	0.577	0.658
RRTI	0.671 (0.550 - 0.791)	0.742	0.583	0.484	0.595
TINN	0.676 (0.558 - 0.795)	0.968	0.333	0.486	0.595
DC	0.722 (0.605 - 0.839)	0.774	0.687	0.615	0.684
AC	0.712 (0.594 - 0.831)	0.774	0.667	0.552	0.646
TP	0.713 (0.599 - 0.827)	0.806	0.604	0.520	0.620
VLF	0.666 (0.544 - 0.788)	0.871	0.458	0.577	0.658
LF	0.718 (0.602 - 0.833)	0.677	0.708	0.619	0.671
HF	0.708 (0.588 - 0.827)	0.613	0.771	0.619	0.671
Model 1	0.765 (0.659 - 0.872)	0.806	0.604	0.586	0.671
Model 2	0.845 (0.761 - 0.930)	0.871	0.750	0.690	0.747

Model 1: All HRV parameters in **Table 2**.

Model 2: Model 1 + Age + BMI + Menopausal status + Mean HR.

## Discussion

This study found that significant increases in certain HRV parameters (such as SDNN and RMSSD) in ECSCC patients were associated with an increase in the incidence of LVSI, and the AUC of the LVSI prediction model established by combining HRV parameters and clinical features reached 0.845. The results indicate that HRV is one of the risk factors for LVSI, and a combined model based on preoperative HRV parameters can be used for predicting the LVSI status in ECSCC patients.

LVSI is an independent risk factor for poor CC prognosis (30). ANS regulation plays a significant role in the tumor microenvironment and is usually associated with tumor occurrence, vascular invasion, and lymph node metastasis (17, 18). Time-domain and frequency-domain analyses of HRV are important methods for evaluating the function of the ANS. They use different mathematical and statistical techniques to quantify changes in heart rate, reflecting the activity levels of the sympathetic nervous system (SNS) and parasympathetic nervous system (PNS) (31). SNS and PNS function antagonistically and work together to regulate unconscious activities of the body, ensuring physiological balance in different environments. Studies have shown that SNS can promote the survival of tumor cells in the initial stage of tumor progression (32), the catecholamines released by SNS can activate the β-adrenergic receptors on tumor cells, regulate gene expression by activating multiple intracellular signaling pathways, thereby enhancing tumor angiogenesis and promoting tumor invasion and metastasis (32). However, PNS has different effects (promoting or inhibiting) on different types of tumors (33). The vagus nerve occupies 75% of the PNS (34) and is believed to regulate the tumor microenvironment by reducing oxidative stress, inhibiting inflammation, and inhibiting the excessive activity of the SNS, thereby improving the immune response of the body to tumor cells (35–38). The acetylcholine (ACh) released by PNS is its main neurotransmitter, which can act on the cholinergic receptor muscarinic 3 (CHRM3), thereby enhancing the activity of the Wnt signaling pathway and promoting the proliferation and migration of tumor cells (39–41). LVSI is widely considered to be a risk factor for tumor metastasis (42). Therefore, the higher rate of tumor metastasis observed in patients with LVSI-positive may be attributed to vagal nerve excitation. RMSSD, DC, and HF mainly reflect vagus nerve activity (43). Our research shows that in ECSCC LVSI-positive, RMSSD, DC, and HF are relatively elevated, indicating enhanced vagus nerve activity, which to some extent confirms this theory. In addition, we found that the absolute value of AC in patients with LVSI-positive was higher than that in patients with LVSI-negative. AC reflects the ability of the heart rate to accelerate in a short period of time and is often used to evaluate the activity of the SNS (25). A higher absolute value of AC indicates that an individual has a stronger physiological adaptability in response to external stimuli and an extremely active state of the SNS (44). VLF activity is also strongly associated with SNS activity, but it is also influenced by various physiological mechanisms (45). Therefore, in LVSI-positive patients, SNS activity is enhanced, as indicated by the increase in the absolute value of AC and VLF, which is consistent with the results of this study. However, since the absolute value of AC and VLF are not standard parameters for evaluating SNS activity, more clinical data and research results need to be combined to further confirm. SDNN, RRTI, TINN, TP, and LF are indicators of overall variability and reflect the joint regulation of the SNS and PNS (31). The body may adapt to physiological changes by enhancing PNS activity during the progression of tumors, especially in the early stages of progression, to support the rapid growth of tumor cells (46). In the tumor microenvironment, the relationship between SNS and PNS is complex and variable, and the activation of PNS may enhance the effects of SNS through a feedback mechanism (47). This may be the reason for the increased variability in LVSI-positive patients in this study.

In clinical practice, accurate identification of LVSI before surgery is of great significance for optimizing treatment plans and improving patient outcomes. In recent years, there have been multiple studies on developing LVSI prediction models based on imaging features. Li et al. (48) developed a nomogram model based on axial T1 contrast-enhanced (CE) MR imaging phenotypic features of 105 CC patients to predict preoperative LVSI status, with an AUC of 0.727 for the test set. Du et al. (49) extracted imaging phenotypic features from T2-weighted imaging of 149 CC patients and combined them with clinical parameters to construct a combined model for predicting preoperative LVSI status. The AUC of the test set was 0.923. Li et al. (50) combined radiogenomics based on PET imaging with tenascin-C (TNC) and cyclooxygenase-2 (COX-2) to establish a machine learning model for predicting LVSI status of CC patients preoperatively, with an AUC of 0.801 for the test set. Malek et al. (51) used the diffusion peak width imaging (DKI) derived parameters of mean diffusion (MD) and mean kurtosis (MK) from 30 CC patients to evaluate the diagnostic accuracy of DKI-derived parameters in identifying LVSI status. The AUCs of the MD and MK parameters were 0.77 and 0.78, respectively. Liu et al. (52) extracted 13 imaging phenotypic features from the MR images of 177 CC patients to develop a multi-parameter magnetic resonance imaging (mpMRI) imaging phenotypic model for predicting LVSI, with an AUC of 0.817 on the test set. All the above studies were conducted on the basis of imaging examinations. Although they were non-invasive prediction method studies, the performance of the models was generally average. This study established a model based on HRV parameters and clinical features, with an AUC of 0.845, a sensitivity of 0.871, a specificity of 0.750, an accuracy of 0.747, and superior predictive performance compared to most MRI models. In contrast, HRV detection is a cost-effective, non-invasive, and clinically feasible predictive tool. With the development of wearable sensor technology, HRV can be captured in daily life (53), making it a promising digital biomarker (54). Based on our research findings, real-time monitoring of HRV can help identify high-risk individuals for CC LVSI in a timely manner, providing a basis for adjusting treatment plans and enabling more precise and personalized medical interventions, thereby improving patient outcomes.

### Limitations

There are some limitations to this study: Firstly, although this study adopted H&E staining combined with IHC detection as the gold standard for evaluating the status of LVSI, existing literature indicates that traditional pathological examination has significant limitations in the diagnosis of LVSI, with insufficient diagnostic stability, manifested as high inter-observer and intra-observer variability, which may affect the accuracy of the model (55). Secondly, this study was conducted as a retrospective analysis and no prospective sample size calculation was performed. However, the effect size calculation (all HRV parameters with Cohen’s d > 0.5, and some indicators > 0.65) and *post hoc* power analysis supported the robustness of the main findings. However, future studies still need to validate the potential of moderate effect parameters through large-sample verification. Finally, the sample data of this study comes from a single center. In the future, multi-center samples should be introduced and multiple machine learning models should be established to further search for models with better performance.

## Conclusion

This study shows that HRV is one of the risk factors for LVSI, and there is a certain correlation between the two, but the mechanism behind this needs further exploration. Meanwhile, an integrated model can be established using HRV parameters of ECSCC patients to predict LVSI status preoperatively. As a non-invasive biomarker, HRV has the potential to provide new technical support for preoperative diagnosis of LVSI in ECSCC patients, providing reference for clinical decision-making and having broad application prospects.

## Data Availability

The raw data supporting the conclusions of this article will be made available by the authors, without undue reservation.
